# Mono-Locus and Pyramided Resistant Grapevine Cultivars Reveal Early Putative Biomarkers Upon Artificial Inoculation With *Plasmopara viticola*

**DOI:** 10.3389/fpls.2021.693887

**Published:** 2021-07-01

**Authors:** Ramona Mihaela Ciubotaru, Pietro Franceschi, Luca Zulini, Marco Stefanini, Domen Škrab, Marcia Denise Rossarolla, Peter Robatscher, Michael Oberhuber, Urska Vrhovsek, Giulia Chitarrini

**Affiliations:** ^1^Department of Agri-Food, Environmental and Animal Sciences, University of Udine, Udine, Italy; ^2^Food Quality and Nutrition Department, Research and Innovation Centre, Fondazione Edmund Mach, San Michele all'Adige, Italy; ^3^Unit of Computational Biology, Research and Innovation Centre, Fondazione Edmund Mach, San Michele all'Adige, Italy; ^4^Genomics and Biology of Fruit Crops Department, Research and Innovation Centre, Fondazione Edmund Mach, San Michele all'Adige, Italy; ^5^Plant Science Department, Federal University of Santa Catarina, Florianópolis, Brazil; ^6^Laimburg Research Centre, Auer, Italy

**Keywords:** downy mildew, metabolomics, mono-locus, pyramided, resistance

## Abstract

One of the most economically important grapevine diseases is Downy mildew (DM) caused by the oomycete *Plasmopara viticola*. A strategy to reduce the use of fungicides to compensate for the high susceptibility of *V. vinifera* is the selection of grapevine varieties showing pathogen-specific resistance. We applied a metabolomics approach to evaluate the metabolic modulation in mono-locus resistant genotypes carrying one locus associated with *P. viticola* resistance (*Rpv*) (BC4- *Rpv1*, Bianca- *Rpv3-1*, F12P160- *Rpv12*, Solaris- *Rpv10*), as well as in pyramided resistant genotypes carrying more than one *Rpv* (F12P60- *Rpv3-1; Rpv12* and F12P127- *Rpv3-1, Rpv3-3; Rpv10*) taking as a reference the susceptible genotype Pinot Noir. In order to understand if different sources of resistance are associated with different degrees of resistance and, implicitly, with different responses to the pathogen, we considered the most important classes of plant metabolite primary compounds, lipids, phenols and volatile organic compounds at 0, 12, 48, and 96 h post-artificial inoculation (hpi). We identified 264 modulated compounds; among these, 22 metabolites were found accumulated in significant quantities in the resistant cultivars compared to Pinot Noir. In mono-locus genotypes, the highest modulation of the metabolites was noticed at 48 and 96 hpi, except for Solaris, that showed a behavior similar to the pyramided genotypes in which the changes started to occur as early as 12 hpi. Bianca, Solaris and F12P60 showed the highest number of interesting compounds accumulated after the artificial infection and with a putative effect against the pathogen. In contrast, Pinot Noir showed a less effective defense response in containing DM growth.

## Introduction

Grapevine was among the first fruit species to be domesticated and today represents one of the most important crops in the world, with an essential role in the economy of many countries. Unfortunately, viticulture is threatened by numerous pathogens causing severe harvest losses. One of the most destructive diseases affecting grapevine is Downy mildew (DM), caused by the biotrophic pathogen *Plasmopara viticola*. DM affects the members of the family *Vitaceae* and in particular the cultivated species *Vitis vinifera* and it can attack all green parts of the vine (leaves, fruits, and shoots in particular) (Buonassisi et al., 2018; Vezzulli et al., 2018). DM infection leads to significant crop losses due to defoliation and to the production of low-quality, deformed or entirely damaged grapes (Yildirim et al., 2019; Nogueira Júnior et al., 2020). The most distinctive signs of infection are the sporangia formation apparent as whitish spots, commonly found on the abaxial surface of the first-formed leaves, which are accompanied by chlorotic spots (known also as oil spots) on the adaxial surface. The sporulation requires humidity > 93% and temperatures of 18–20°C and it can be observed on the abaxial side of the leaf and the surface of tendrils, inflorescence, and young berries (Buonassisi et al., 2018).

The application of large amounts of fungicides is the most diffused strategy to control DM, this practice, however, is not only expensive and in conflict with the requirements for sustainable and environment-friendly agriculture, but also promotes the emergence of fungicide-resistant strains (Buonassisi et al., 2018; Merdinoglu et al., 2018; Fröbel and Zyprian, 2019; Yildirim et al., 2019). A possible alternative to the use of fungicides is the valorization of the interspecific hybrids of *V. vinifera* with resistant genotypes from *Muscadinia*, several wild North American and Asian *Vitis* species which have been found resistant against *P. viticola* (Buonassisi et al., 2017; Merdinoglu et al., 2018; Vezzulli et al., 2018; Fröbel and Zyprian, 2019).

The resistance response to *P. viticola* is given by quantitative trait loci (QTLs) named *Rpv* (i.e., resistance to *P. viticola*). To date, 27 quantitative trait loci (QTL) have been identified in wild *Vitis* species and a descriptive list of them is available online (www.vivc.de/data on breeding and genetics/ Table of loci for Traits in Grapevine) (Bellin et al., 2009; Bove et al., 2019; Eisenmann et al., 2019; Vezzulli et al., 2019; Maul et al., 2020; Nogueira Júnior et al., 2020). The protection offered by these resistance genes (*R* genes) can be overcome by virulent strains of the pathogen, particularly in the genotypes carrying one *Rpv* gene (Peressotti et al., 2010; Merdinoglu et al., 2018; Fröbel et al., 2019). To avoid such resistance breakdowns a longer-lasting disease resistance is required. A possible strategy is pyramiding resistance, by accumulating several resistant genes in the same variety to create a durable disease resistance (Merdinoglu et al., 2018; Stam and McDonald, 2018). The study of varieties with different resistance genes can help us explore the mechanisms of resistance in *P. viticola*-grapevine interactions. Thus, this study initially screened four cultivars with mono-locus resistance (BC4, Bianca, F12P160 and Solaris) and subsequently two cultivars with pyramided resistance (F12P60 and F12P127).

Grapevine cultivar Bianca is a Bouvier and Villard Blanc hybrid, created in 1963 at the Kölyuktetö viticulture research facility in Hungary. Its resistance is given by the *Rpv3-1* locus located in chromosome 18 (Bellin et al., 2009). The cultivar Solaris was obtained by crossing the variety Merzling (*Rpv3-3*) with Gm6493 (*Rpv10*). It was created at the Geisenheim grape-breeding Institute (Germany) and is a carrier of resistance locus *Rpv10* that maps to chromosome 9 (Schwander et al., 2012). Both varieties are officially registered for use in wine production (http://www.vivc.de/). The resistance of the F12P160 genotype is explained by the *Rpv12* locus, located in chromosome 14 (Venuti et al., 2013). The cultivar BC4 was created in 2017 at INRA (France) as a cross between *Muscadinia* (*Rpv1*) X Regent (*Rpv3-1*). The *Rpv1* locus is responsible for its resistance and it maps to chromosome 12 (Merdinoglu et al., 2003). None of the two hybrids are officially registered for use in wine production. The latest cultivars, F12P60 and F12P127, are two pyramided hybrids created at Fondazione Edmund Mach (Italy). *Rpv3-1* and *Rpv12* are responsible for the resistance in cultivar F12P60 and they map to chromosomes 18 and 14, respectively (Bellin et al., 2009; Venuti et al., 2013). The resistance loci *Rpv3-1, Rpv3-3*, and *Rpv10* map to the chromosomes 18 and 9 and are engaged in the resistance of the cultivar F12P127 (Bellin et al., 2009; Di Gaspero et al., 2012; Schwander et al., 2012). Both varieties are not yet registered for cultivation.

Information about the different behavior of resistant and susceptible varieties coming from several cultivars is useful to understand the protection mechanisms involved in resistance to *P. viticola*. The plasticity of the plants in response to the pathogen is probably associated with the modulation of several classes of primary and secondary metabolites. For this reason, metabolomics is the most suitable approach in exploring the interaction between the grapevine and *P. viticola* and in extending the current knowledge about the perturbations of a wide range of molecules after biotic stress. To date, metabolomics studies have focused on several aspects: the differences between grapevine cultivars in berry composition in some cases (Mulas et al., 2011; Degu et al., 2014; Teixeira et al., 2014; Bavaresco et al., 2016), and the identification of metabolite changes in infected leaves in others (Ali et al., 2012). Some works focused on the metabolomic profiling of grapevine tissues infected with DM (Figueiredo et al., 2008; Ali et al., 2009; Buonassisi et al., 2017) and on metabolite changes due to the mono-locus resistance mechanism (Chitarrini et al., 2017, 2020). However, there is not yet a full description of which metabolites play a key role in resistance in the pyramiding resistance cultivars. This suggests the need to investigate further to identify the biomarkers of the defense response in resistant varieties.

In this study, we chose to examine first the reaction of primary and secondary metabolism of genotypes with mono-locus resistance against DM, and then we extended our investigation to the analysis of pyramided resistance genotypes. Among the hundreds of compounds identified, we decided to focus on those metabolites (not stilbenes and stilbenoids) that showed significant accumulation in resistant vs. susceptible genotypes over the course of the infection, and that can therefore be identified as putative markers of resistance. Within the class of stilbenes and stilbenoids we decided to investigate not only the putative markers of resistance but also the markers of infection. The aim was to find previously unreported biomarkers of resistance, which are expected to pave the way for a better understanding of the different resistance mechanisms that underlie the hybrids-pathogen interaction affecting the *Vitis* species. All genotypes in the study were observed over 2 consecutive years and examined with a metabolomics approach for primary and secondary metabolism at 0, 12, 48, and 96 h post-inoculation. The assessment of the resistance level after artificial inoculation on leaves was carried out using the OIV-452 method (Supplementary Table 1).

## Materials and Methods

### Plant Material and Artificial Inoculation

Grapevine plants with genotypes having different degrees of resistance to DM and one with a susceptible genotype were used in this study. The mono-locus resistance genotypes consisted of the varieties BC4, Bianca, F12P160 and Solaris whereas the pyramided resistance genotypes were F12P60 and F12P127 (Table 1). All the grapevine plants were grown in pots in controlled conditions in the Fondazione Edmund Mach grape germplasm collection located in San Michele all‘Adige (Trento), Italy (46^0^ 12′ 0″ N, 11^0^ 8′ 0″ E). The mono-locus resistance experiment was conducted in the 2 consecutive years 2016 and 2017; while the pyramided resistance experiment was conducted in the 2 consecutive years 2017 and 2018. For each experiment, the susceptible variety Pinot Noir was used as control genotype (Table 1).

**Table 1:** The genotypes used in this study, their source of resistance and their associated resistance-related loci (*Rpv*) with their references.

**Genotypes**		**Resistance related loci (*****Rpv*****)**			**References**
		**Downy mildew**	**Preliminary leaf resistance level**	**Source of resistance**	
Mono-locus resistance	BC4	*Rpv1*	Resistant	*M. rotundifolia*	Merdinoglu et al., 2003
	Bianca	*Rpv3-1*	Resistant	*V. rupestris*	Bellin et al., 2009
	F12P160	*Rpv12*	Resistant	*V. amurensis*	Venuti et al., 2013
	Solaris	*Rpv10*	Resistant	*V. amurensis*	Schwander et al., 2012
Pyramided resistance	F12P60	*Rpv3-1; Rpv12*	Resistant	*V. rupestris*	Bellin et al., 2009; Venuti et al., 2013
				*V. amurensis*	
	F12P127	*Rpv3-1, Rpv3-3; Rpv10*	Resistant	*V. rupestris*	Bellin et al., 2009; Di Gaspero et al., 2012; Schwander et al., 2012
				*V. amurensis*	
Control	Pinot Noir	–	Susceptible	–	

**Figure 1:**
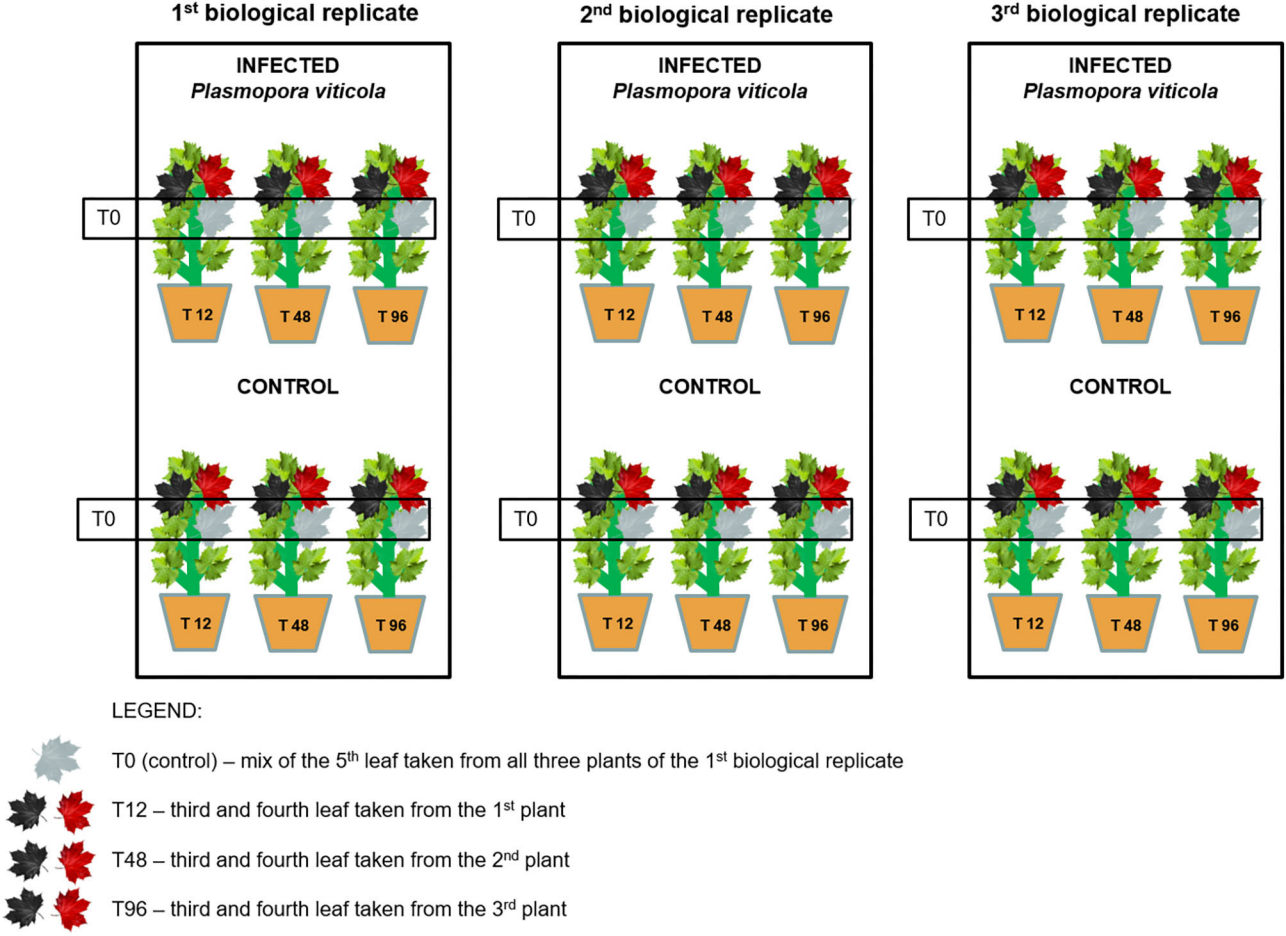
Experimental design and randomization scheme.

During the experiment, the healthy plants (*n* = 18 per variety) were divided into two homogeneous groups (control and inoculated); the plants in the same group were further divided into three groups, each one representing one biological replicate (Figure 1). Plants were artificially infected with spores of the pathogen in the greenhouse. The inoculum was collected each year in late spring/early summer from naturally infected plants of the same untreated vineyard (grape cultivar: Pinot Noir) and was characterized by a mix of strains. Grapevine plants were inoculated by spraying the sporangial suspension at the rate of 1x10^6^ sporangia/mL on the lower surface of all leaves of plants, whereas the control plants were sprayed using milliQ water. Plants were kept in the greenhouse at a controlled temperature of 21°C and over 80% of relative humidity until the sampling. Leaves were sampled at four time points following a randomization scheme at 0, 12, 48, and 96 h post-inoculation/mock (Figure 1). Three biological replicates were sampled at each time point. Each sample was ground under liquid nitrogen and stored at −80°C until the extractions. The OIV-452 score was evaluated at 7 days post-inoculation on the first six fully expanded leaves (Supplementary Table 1) to assign a resistance score to *P. viticola* (leaves): 1 = very low 3 = low 5 = medium 7 = high 9 = very high or total. At the same time the Hypersensitive Response (HR) identified by the necrosis spots was evaluated.

### Extraction Procedures and Analysis of Compounds

#### Primary Compounds

Primary compounds were extracted from 100 mg of fresh leaves and then subjected to derivatization using methoxamine hydrochloride in pyridine to inhibit the cyclization of reducing sugars and then with N-methyl-N-trimethylsilyl-trifluoroacetamide with 1% trimethylchlorosilane for trimethylsilylation following the Chitarrini et al. (2017) procedure. The derivatized extract was then injected for GC/MS analysis using a Trace GC Ultra with a fused silica RXI-5-Sil MS w/Integra Guard (30 m × 0.25 mm × 0.25 μm) column, combined with mass spectrometer TSQ Quantum GC (Thermo Electron Corporation) following the Chitarrini et al. (2017) parameters.

#### Volatile Compounds

Volatile compounds were measured using a solid phase micro-extraction starting from 100 mg of fresh leaves and following the method of Chitarrini et al. (2017). Gas chromatography separation was done using a Trace GC Ultra gas chromatograph with a fused silica Stabilwax-DA column (30 m × 0.25 mm × 0.25 μm) (Restek Corporation) coupled to a Quantum XLS mass spectrometer (Thermo Electron Corporation) following the parameters of Matarese et al. (2014).

#### Lipidic Compounds

Lipid compounds analysis was done according to Della Corte et al. (2015) following the sample preparation described by Chitarrini et al. (2017). One hundred mg of fresh leaves were extracted using 0.3 mL of methanol; 0.6 mL of chloroform containing butylated hydroxyl toluene (500 mg/L); 0.25 mL water and then with 0.4 mL of chloroform containing butylated hydroxyl toluene (500 mg/L)/methanol/water 86:14:1 v/v/v; the combined lower lipid-rich layer was evaporated to dryness under N_2_ and the samples were re-suspended in 300 μl of acetonitrile/isopropanol/water (65:30:5 v/v/v/). Samples were injected into a UHPLC Dionex 3000 (Thermo Fisher Scientific) with RP Ascentis column (15 cm × 2.1 mm; 2.7 μm C18) following a 30 min multi-step gradient coupled with an API 5500 triple-quadrupole mass spectrometer (Applied Biosystems/MDS Sciex) (Della Corte et al., 2015).

#### Phenolic Compounds

The phenolic compounds were extracted from 100 mg of fresh leaves using 0.4 mL of chloroform and 0.6 mL of methanol:water (2:1); the extraction was repeated by adding 0.6 mL of methanol and water (2:1 v/v) and 0.2 mL of chloroform according to Vrhovsek et al. (2012) with some modifications, previously applied by Chitarrini et al. (2017). The aqueous-methanol phase of two extractions was collected, combined, and evaporated to dryness under N_2_. Samples were re-suspended in 500 μl of methanol: water (1:1 v/v) and injected in a Waters Acquity UPLC system (Milford) with a Waters Acquity HSS T3 column (10 mm × 2.1 mm; 1.8 μm) coupled with a Xevo triple-quadrupole spectrometer (Waters) following Vrhovsek et al. (2012).

### Data Processing and Statistical Analysis

Data processing of primary and volatile compounds was performed using the software “Xcalibur” (version 4.0), whereas “Analyst” (version 1.7) and “MassLynx” (version 4.1) were used for processing lipids and phenols, respectively.

Lipid, phenols and primary compounds were identified using reference standards, retention time, quantifier and qualifier ion, and quantified using their standard calibration curves as mg/kg of fresh leaves. Volatile organic compounds were identified in the mass spectral database NIST MS Search 2.3 and results were semi quantified as the equivalent of the internal standard (1-heptanol) and expressed as μg/kg of fresh leaves.

Statistical analysis and visualization were performed with R (R Core Team, 2020) relying on the following packages: tydiverse (Grolemund et al., 2019; Wickham et al., 2019) and egg (Baptiste, 2019) for data handling, manipulation and visualization; emmeans packages (Russell, 2020) for marginal means estimations; effsize for the effect size calculation (Sawilowsky, 2009; Torchiano, 2020). Logarithmic transformation was used to correct for the expected non-normality of metabolomics data. The average effect of each year was subtracted for each metabolite/genotype, to compensate for the expected year-to-year variability in the overall metabolic response. A linear modeling approach was used for each metabolite/genotype to assess the effects of time and artificial inoculationt (inoculated and non-inoculated). Cohen's d was used to estimate the size of the metabolic modulation induced by the pathogen inoculation for each time point. A metabolite was considered significantly perturbed if its concentration in the inoculated samples was significantly different from the control plants at least at one time point (uncorrected *p* < 0.05).

## Results

### Dynamics of Metabolic Perturbations in Plant Defense Mechanism

In the 2 years considered, 264 compounds were identified in leaf samples under investigation. Among these, we quantified 175 compounds belonging to several classes: organic acids (29), amino acids (17), amines and others (12), sugars (25), benzoic acids derivatives (6), coumarins (3), dihydrochalcones (1), flavan-3-ols (11), flavanones (2), flavones (4), flavonols (15), phenylpropanoids (5), stilbenes and stilbenoids (13), fatty acids (15), glycerolipids (4), glycerophospholipids (2), prenols (1), sphingolipids (1), sterols (2) and other phenols (7). We semi-quantified 89 volatile organic compounds: volatile acids (5), alcohols (14), aldehydes (13), benzenoids (6), esters (3), hydrocarbons (1), other volatiles (6), fatty acids (2), benzofurans (1), terpenoids (10), terpenes (10), ketones (4) and unknown volatiles (14). In Supplementary Table 2 the concentrations of VOCs (sheet 1) lipids (sheet 2) and polyphenols (sheet 3) identified as putative markers of resistance following the criteria described in section Putative Biomarkers of Resistance to *Plasmopara viticola* and The Effect of Pathogen Inoculation have been reported together with stilbenes and stilbenoids involved in the response to the infection and fight against the pathogen (sheet 3; see section Putative Biomarkers of Resistance to *Plasmopara viticola* and Stilbenes and Stilbenoids as Markers) for each genotype and for each year (Supplementary Table 2).

### Putative Biomarkers of Resistance to *Plasmopara viticola*

**Figure 2:**
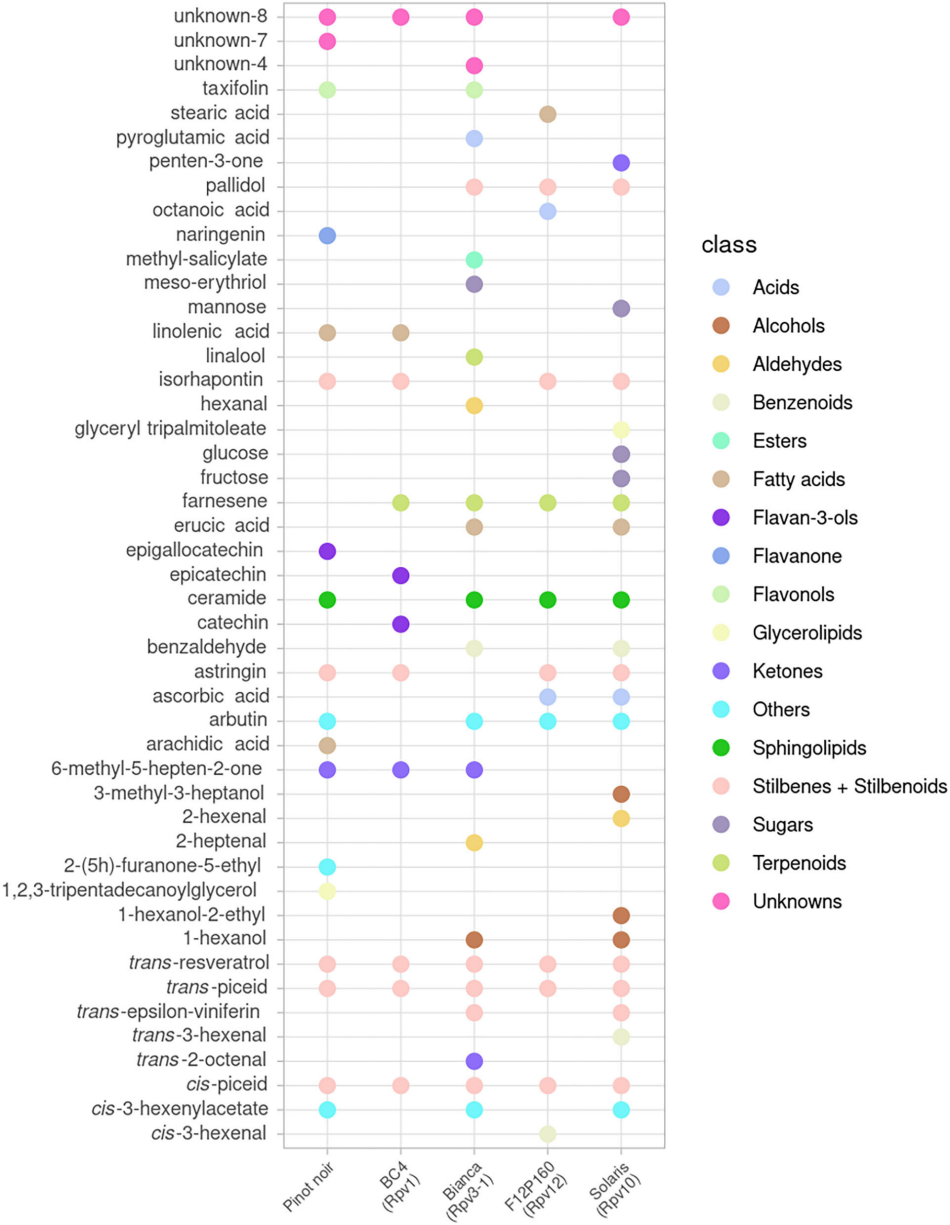
Metabolites significantly modulated by the infection in at least one-time point for mono-locus resistant genotypes (BC4, Bianca, F12P160, Solaris) and for the susceptible Pinot Noir. All time points were considered in the 2 years of data analysis (2016–2017) and the color of each metabolite identifies the different chemical classes.

**Figure 3:**
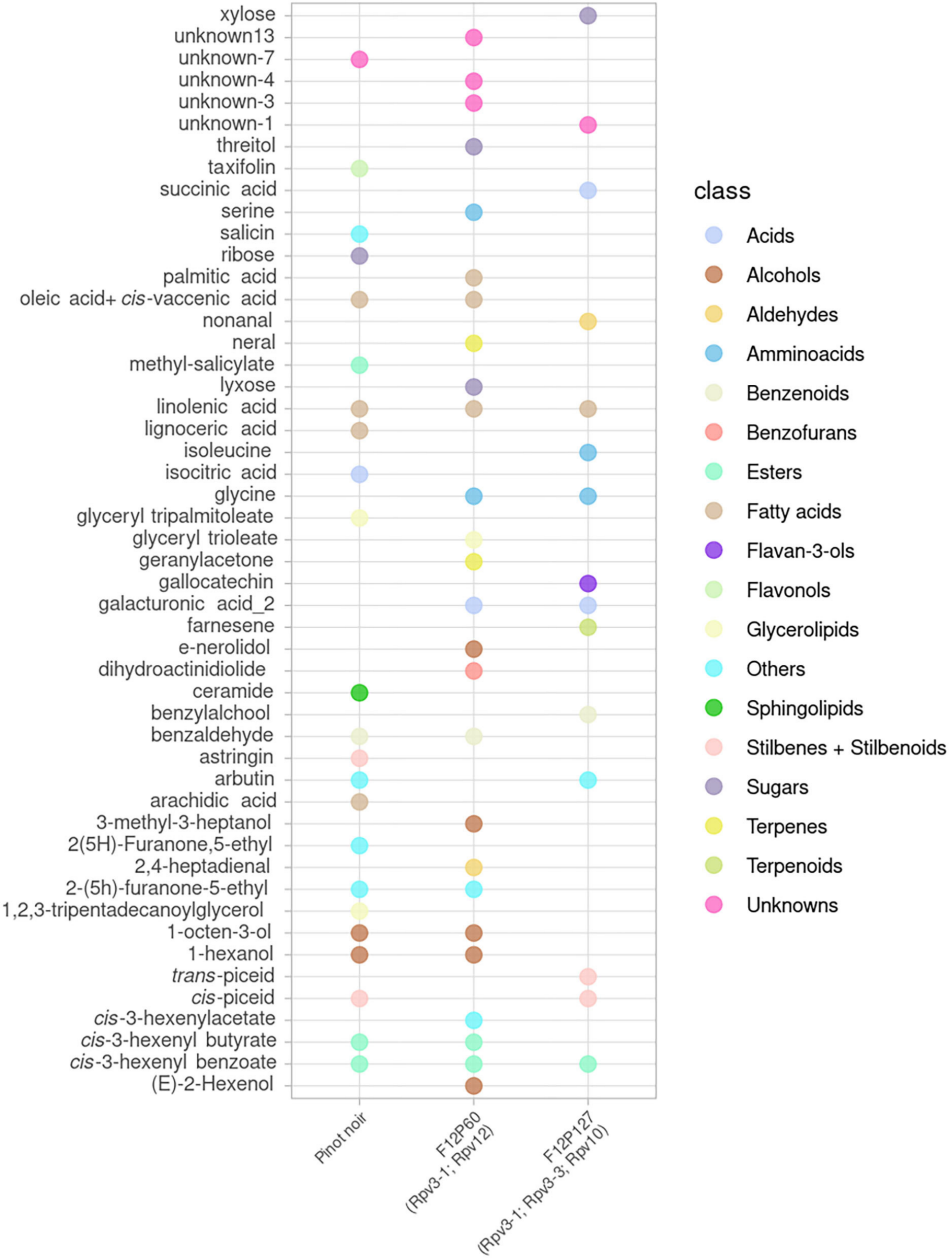
Metabolites significantly modulated by the infection in at least one time point for the pyramided resistant genotypes (F12P60, F12P127) and for the susceptible Pinot Noir. All time points were considered in the 2 years of data analysis (2017–2018) and the color of each metabolite identifies the different chemical classes.

A global view of the metabolites that showed a significant effect after inoculation (*p* < 0.05 in at least one time point) is presented for the mono-locus resistant genotypes (BC4, Bianca, F12P160, Solaris) in Figure 2 and for the pyramided resistant genotypes (F12P60, F12P127) in Figure 3.

In the plots, the dots indicate in which genotype(s) each metabolite was showing a significant difference in the inoculated vs. non-inoculated samples at least at one time point. This global visualization highlights that the resistant varieties Bianca, Solaris and F12P60 are showing a higher number of modulated metabolites. On the other hand, BC4 and F12P127 seem to show a more limited response to inoculation. The OIV-452 score was evaluated in the experiments (Supplementary Table 1) showing a very high degree of resistance for Bianca, F12P160, F12P60 and F12P127 (OIV-452 = 9); a high level for Solaris (OIV-452 = 7), medium for BC4 (OIV-452 = 5) and very low for Pinot Noir (OIV-452 = 1). At the same time, the hypersensitive response (HR) taking into account the necrosis was evaluated following the OIV-452 score. HR response was absent in Pinot Noir, medium in F12P160 and Bianca and high in BC4 and Solaris. For the pyramided genotypes, F12P127 was characterized by an high level of HR response, whereas the HR response was absent in F12P60 (Supplementary Table 1).

Figures 2, 3 clearly show that the interaction with the pathogen profoundly alters the plant metabolism, and some of the metabolites appear modulated after the artificial inoculation in both resistant genotypes and the susceptible Pinot Noir.

In order to pinpoint the most promising compounds, we defined the following for potential resistance biomarkers:

for metabolites excluding stilbenes and stilbenoids:

1. the metabolite was showing a significant modulation only in the resistant genotypes and, in addition, it was showing a large positive modulation (effect size d > 1) at the last two time points.

for stilbenes and stilbenoids:

1. the metabolite was showing a significant modulation only in the resistant genotypes and, in addition, it was showing a large positive modulation (effect size d > 1) at the last two time points (see section Stilbenes and Stilbenoids as Markers);
2. if modulated also in Pinot Noir, the metabolite was showing an effect size with a delta d > 1 compared with Pinot Noir (see section Stilbenes and Stilbenoids as Markers).

In the case of non-stilbenoids, we acknowledge that the magnitude and the timing of the accumulation of a compound could be important in characterizing the response of the plant to the pathogen attack (Pezet et al., 2004; Chitarrini et al., 2017), but the presence of a significant modulation also in Pinot Noir suggests that this metabolite is actually associated with infection. The second part of the first criterion (d > 1 in the last two time points), instead, stemmed from the hypothesis that the presence of the pathogen in the inoculated leaves was the main cause for the accumulation of the metabolites over time. In the case of stilbenoids, a more liberal criterion was applied since this class of compounds is known to hold a prominent role in the response of *V. vinifera* to pathogen infection; for these reasons we considered also those compounds with an effect size in the inoculated conditions with a delta d > 1 compared with Pinot Noir.

### The Effect of Pathogen Inoculation

The previous criteria led to the identification of 20 compounds, excluding the stilbenes and stilbenoids class (discussed in section Stilbenes and Stilbenoids as Markers), as putative biomarkers of resistance belonging to the plant primary metabolism: fatty acids (4) and secondary metabolism: flavan-3-ols (1), alcohols (4), aldehydes (2), benzenoids (1), benzoic acid esters (1), terpenoids (4), esters (1), and unknown volatiles (2) (Table 2). The concentrations of these compounds of interest are reported in Supplementary Table 2.

**Table 2:** Potential biomarkers among all metabolite classes except stilbenes and stilbenoids as identified by the selection criterion—modulation only in the resistant genotypes (d > 1).

**Class of the compounds**	**Compounds**	**GENOTYPES**					
		**Mono-locus resistance**				**Pyramided resistance**	
		**BC4 (*Rpv1*)**	**Bianca (*Rpv3-1*)**	**F12P160 (*Rpv12*)**	**Solaris (*Rpv10*)**	**F12P60 (*Rpv3-1; Rpv12*)**	**F12P127 (*Rpv3-1, Rpv3-3; Rpv10*)**
Fatty acids	erucic acid		•				
	oleic acid + *cis*-vaccenic acid					•	
	palmitic acid					•	
	stearic acid			•			
Flavan-3-ols	Epicatechin	•					
Alcohols	1-hexanol		•		•	•	
	1-hexanol-2 ethyl				•		
	(*E*)-2 hexenol					•	
	1-octen-3-ol					•	
Aldehydes	2-hexenal				•		
	nonanal						•
Benzenoids	benzaldehyde		•		•	•	
Benzoic acid esters	methyl salicylate		•				
Terpenoids	farnesene	•	•	•			•
	linalool		•				
	(*E*)-nerolidol					•	
	neral					•	
Esters	*cis*-3-hexenyl benzoate					•	
Unknowns VOCs	unknown 4					•	
	unknown 13					•	

In order to discuss the strength of the modulation induced by the pathogen, the effect size (Cohen d) was calculated for each putative biomarker and for each time point (0, 12, 48, 96 hpi). According to the study of Sawilowsky (2009), the “d” values are associated with an effect size which can vary from a very small (d = 0.01) to a huge effect (d = 2.0). The “d” values of the identified putative biomarkers and their associated effect size are being presented in the Supplementary Table 3.

#### BC4

**Figure 4:**
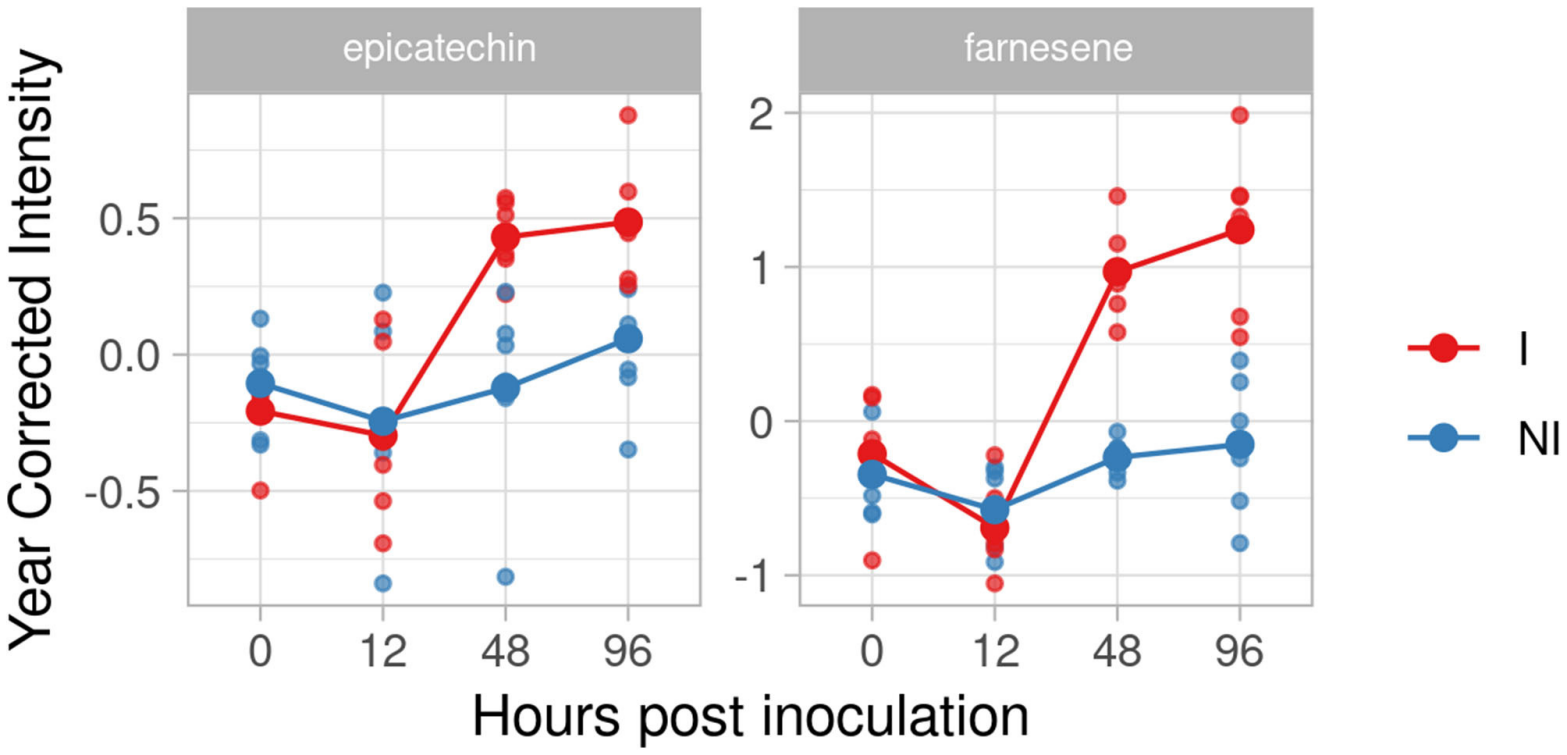
Graphs for specific putative biomarkers of resistance to *Plasmopara viticola* in inoculated (Red) and not inoculated (Blue) BC4 genotype. Values of the 2 years are reported after subtracting the year's effect. I, inoculated; NI, not inoculated.

In the resistant genotype BC4 we identified two compounds as putative biomarkers; one phenol, epicatechin, and one volatile, farnesene. Catechin and epicatechin have been recently identified as discriminatory factors, with a significantly higher amount in resistant/partial resistant plants (Maia et al., 2020). In our experiment, the effect size of epicatechin strongly grew at 48 and 96 hpi (1.99 and 1.64). Farnesene, instead, showed a higher and rapid accumulation after 12 hpi with high d values at 48 and 96 hpi (5.15 and 2.78) (Figure 4).

#### Bianca

**Figure 5:**
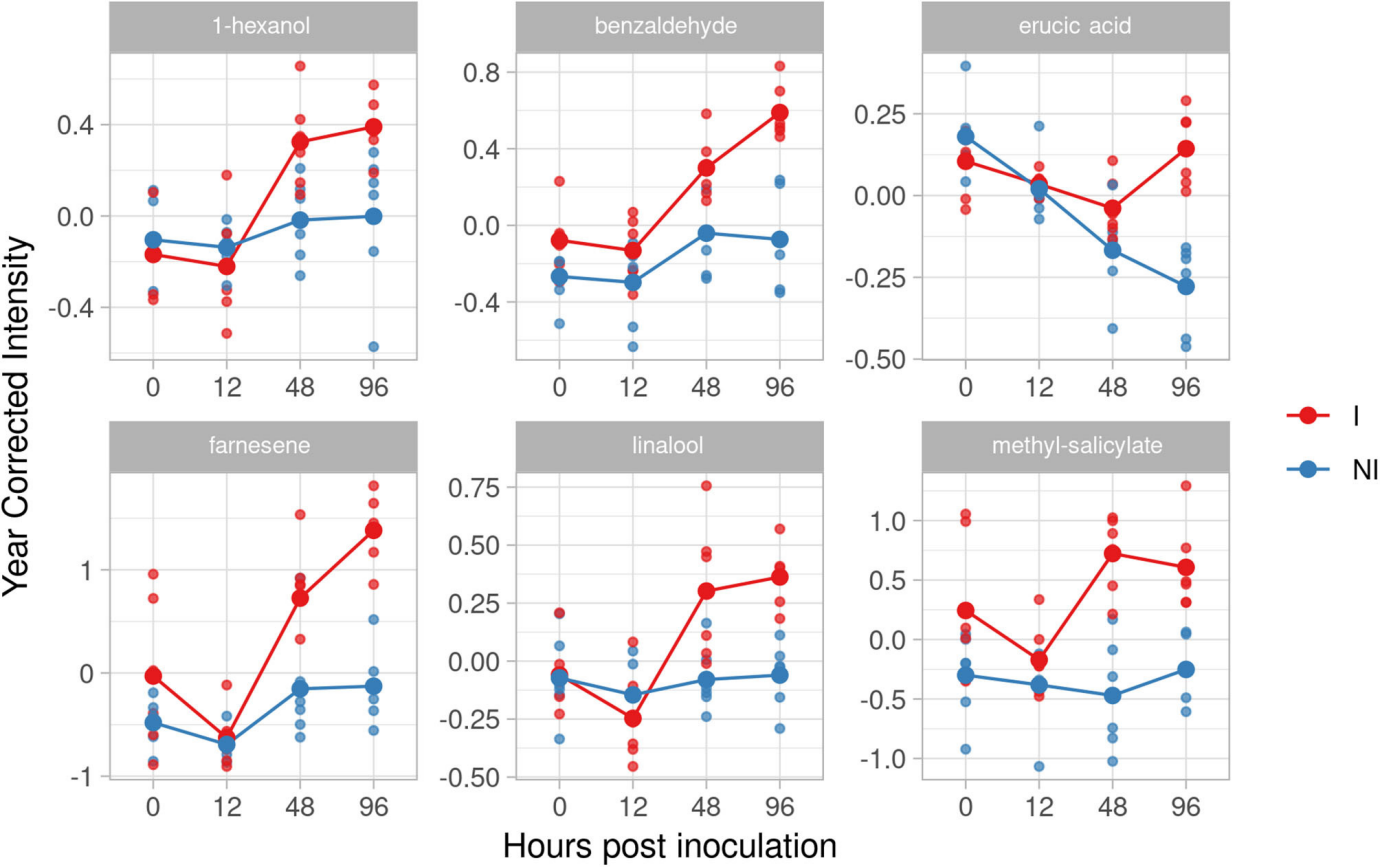
Graphs for specific putative biomarkers of resistance to *Plasmopara viticola* in inoculated (Red) and not inoculated (Blue) Bianca genotype. Values of the 2 years are reported subtracting the year's effect. I, inoculated; NI, not inoculated.

In the resistant genotype Bianca, six VOCs have been identified as potential biomarkers: 1-hexanol, erucic acid, benzaldehyde, farnesene, linalool, methyl-salicylate (Figure 5). In five of them we found an accumulation with a positive effect at both 48 and 96 hpi. The effect size of inoculation for 1-hexanol increased at 48 and 96 hpi, where it reached a positive effect (1.77 and 1.53, respectively). Linalool started increasing at 48 hpi (1.60), reaching a positive effect (3.05) at 96 hpi. Farnesene increased showing an effect size of 1.56 at 48 hpi and reaching a huge effect size of 4.23 at 96 hpi. The last two significant compounds of this resistant genotype, benzaldehyde, and methyl salicylate kept a positive effect immediately after the inoculation reaching an effect size at 48 and 96 hpi (benzaldehyde 1.66 and 3.16 at 48 and 96 hpi; methyl salicylate 2.96 and 2.61 at 48 and 96 hpi). Benzaldehyde was present also in F12P60 and Pinot Noir for 2017–2018, whereas methyl salicylate was detected in Pinot Noir for 2017–2018. Since in these cases the effect of the inoculation was much smaller, they remain putative biomarkers of resistance as initially assumed. Erucic acid reaches a peak with positive effect at 96 hpi (3.32).

#### F12P160

**Figure 6:**
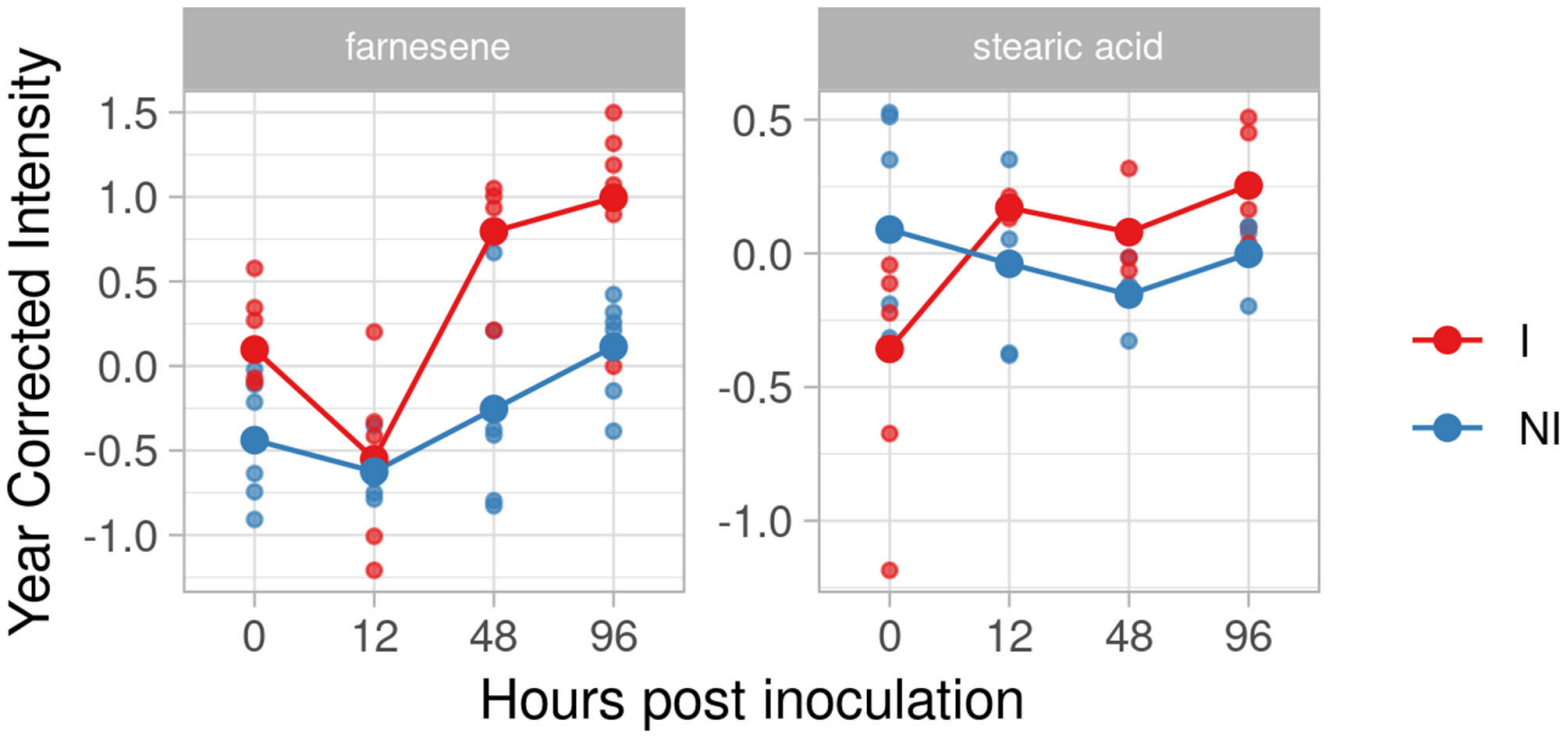
Trend graph over time of putative biomarkers of resistance to *Plasmopara viticola* in F12P160 genotype inoculated (Red) and not inoculated (Blue). Values of the 2 years are reported subtracting the year's effect.

For the resistant genotype F12P160, we identified farnesene and stearic acid in our inclusion criteria list. Figure 6 highlights the interesting accumulation of farnesene with an increase of the effect size at 48 and 96 hpi (2.12 and 2.03, respectively).

#### Solaris

**Figure 7:**
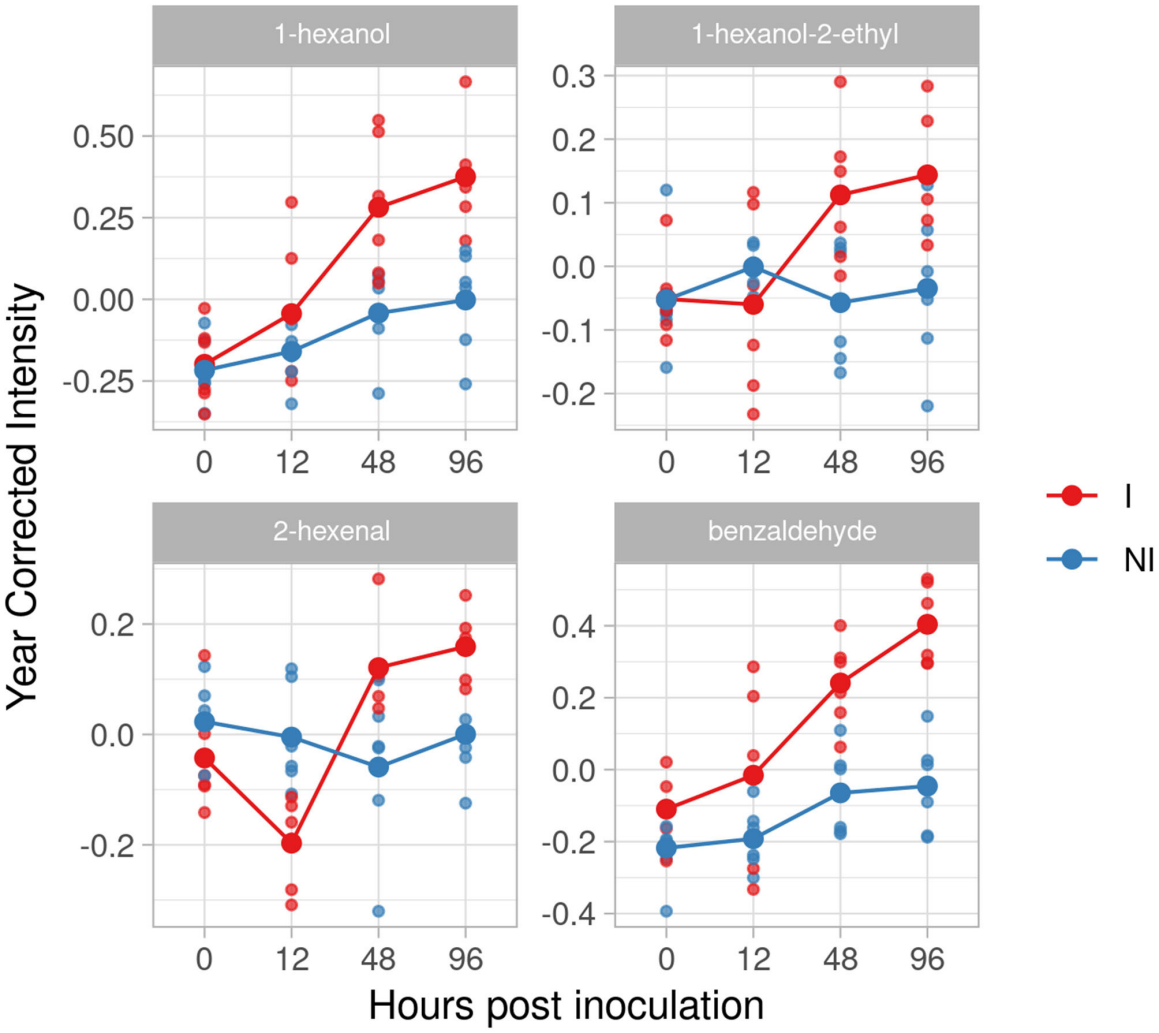
Graphs for specific putative biomarkers of resistance to *Plasmopara viticola* in inoculated (Red) and not inoculated (Blue) Solaris genotype. Values of the 2 years are reported subtracting the year's effect.

In the resistant genotype Solaris, we identified four compounds: 1 hexanol, 1-hexanol-2-ethyl, 2-hexenal and benzaldehyde (Figure 7). All the four metabolites are accumulated at 48 hpi with a peak at 96 hpi and an effect size of 2.33, 1.61, 1.97 and 3.65.

#### F12P127

**Figure 8:**
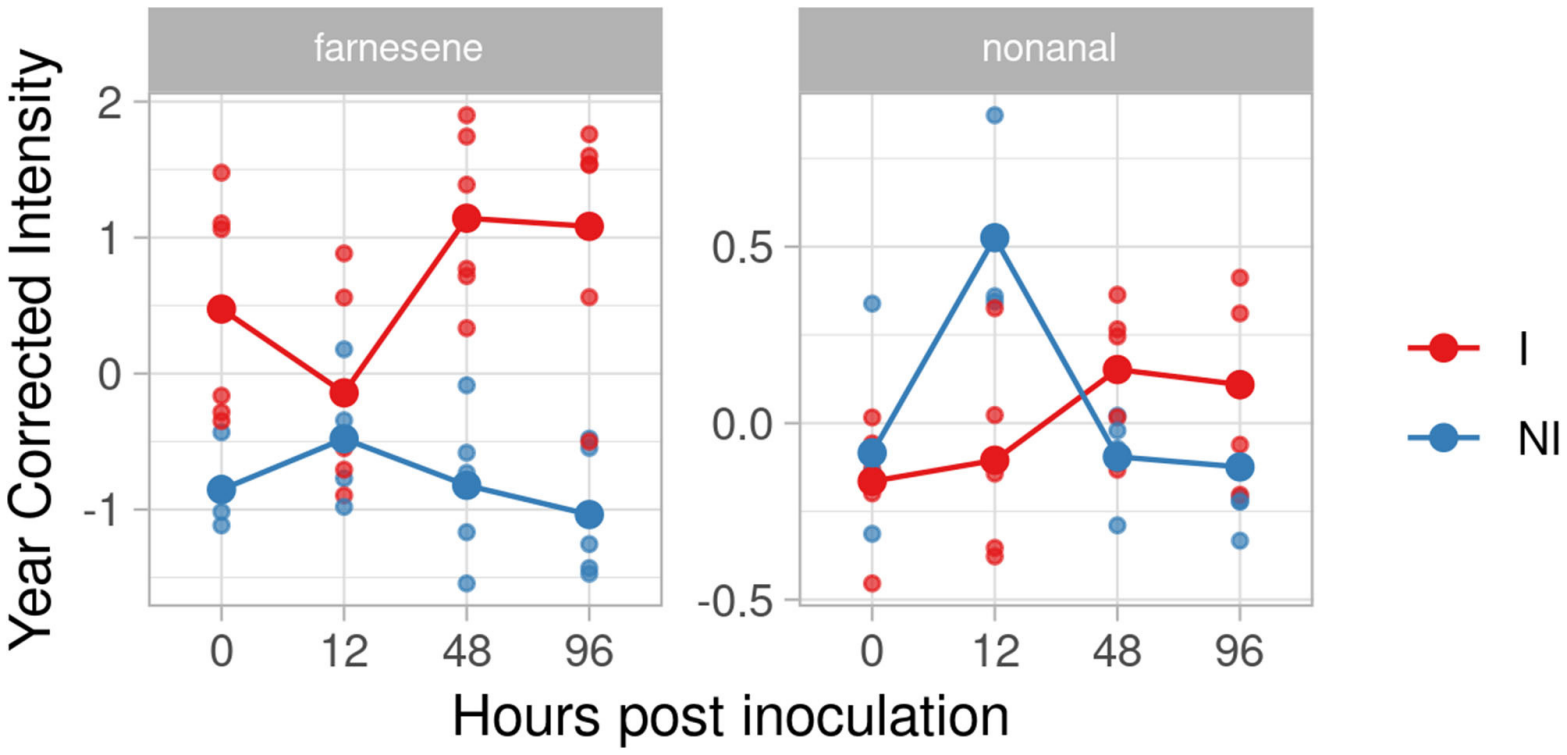
Graphs for specific putative biomarkers of resistance to *Plasmopara viticola* in inoculated (Red) and not inoculated (Blue) F12P127 genotype. Values of the 2 years are reported subtracting the year's effect.

The pyramided genotype F12P127 revealed two compounds in the inclusion criteria list, farnesene and nonanal (Figure 8). Farnesene was accumulated at 48 and 96 hpi with an effect size of 3.28 and 2.88, while nonanal showed an unclear trend among the time with an effect size of 1.48 and 1.10 at 48 and 96 hpi.

#### F12P60

**Figure 9:**
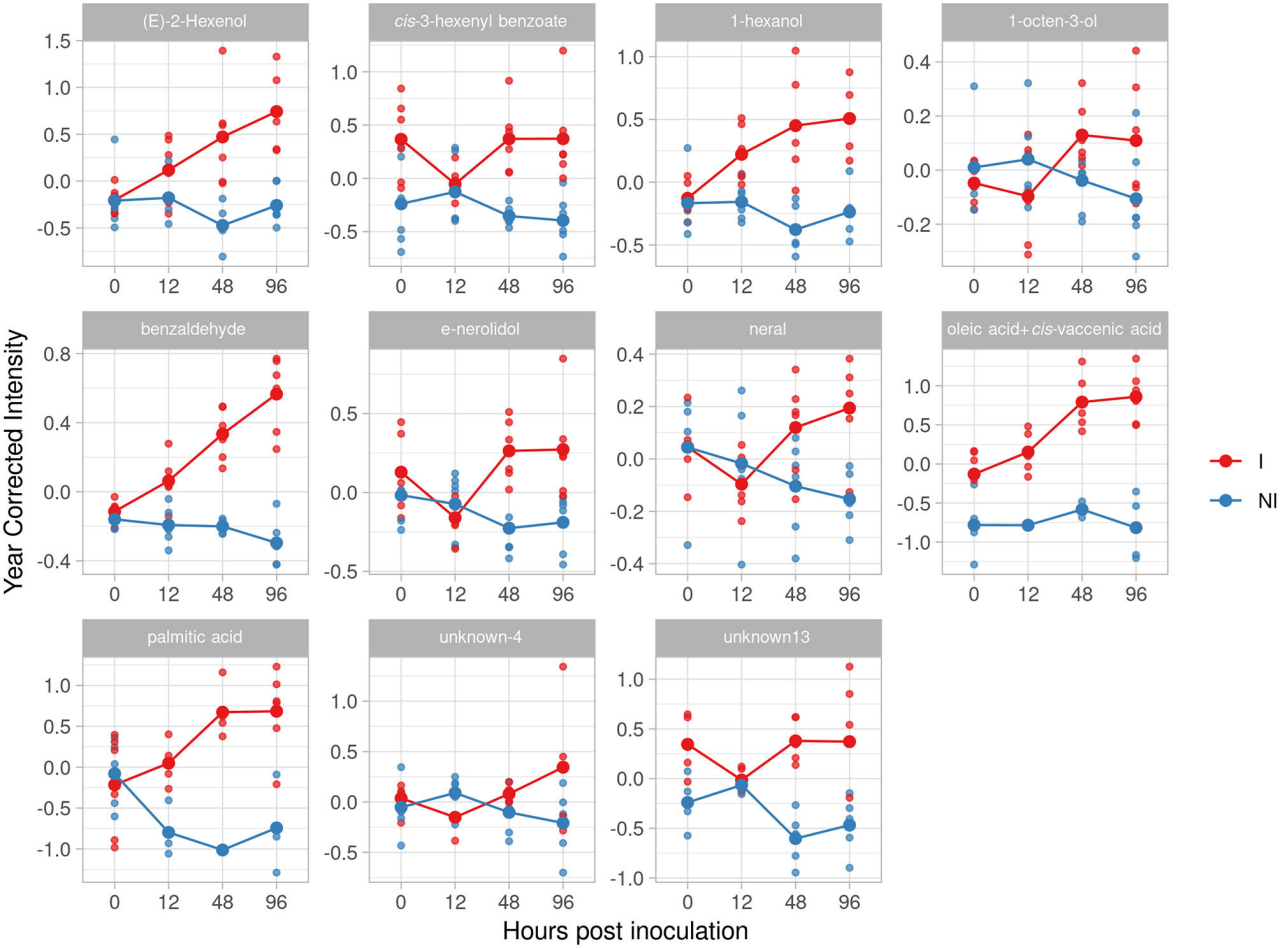
Graphs for specific putative biomarkers of resistance to *Plasmopara viticola* in inoculated (Red) and not inoculated (Blue) F12P60 genotype. Values of the 2 years are reported subtracting the year's effect.

In F12P60 pyramided genotype, we identified eleven potential biomarkers (Table 2) in total. Benzaldehyde, as for F12P127 and Bianca genotypes, increased reaching an effect size of 4.92 and 4.76 at 48 and 96 hpi. Similar trends were found for (*E*)-2-hexenol (2.97 at 96 hpi) and 1-hexanol (2.74 at 96 hpi). The two terpenoids (*E)*-nerolidol and neral are accumulated after 24 hpi, with a peak at 48 hpi for (*E*)-nerolidol (2.17) and at 96 hpi for neral (2.27), respectively. Finally, we found a lipid compounds accumulation: oleic acid+*cis-*vaccenic and palmitic acid have an accumulation trend over time with an effect size of 7.01 at 48 hpi for palmitic aid and 4.44 and 4.5 for oleic acid+*cis-*vaccenic at 48 and 96 hpi (Figure 9).

### Stilbenes and Stilbenoids as Markers

Following the described criteria (see section Putative Biomarkers of Resistance to *Plasmopara viticola*), we found six significant compounds (Table 3); among them, pallidol and *trans*-epsilon-viniferin were the only compounds not modulated in Pinot Noir (for the concentrations seen Supplementary Table 2 sheet 3). Pallidol reached the first criteria for stilbenes and stilbenoids in Bianca (effect size of 3.45 at 96 hpi), F12P160 (1.30 at 48 hpi and 3.07 at 96 hpi), and Solaris (2.02 at 48 hpi and 6.47 at 96 hpi); looking at the trend figures we found a comparable reaction in BC4 without a significant effect size (Supplementary Figure 1). The same situation is reported for *trans*-epsilon-viniferin, that reached the selected criteria in Bianca (3.17 at 96 hpi) and Solaris (1.19 at 48 hpi and 6.61 at 96 hpi) and reacted with a similar trend in F12P160 and BC4 but not with a significant effect size (Supplementary Figure 1).

**Table 3:** Potential biomarkers among stilbenes and stilbenoids as identified by the selection criterion.

**Compounds**	**Genotypes**						
	**Susceptible**	**Mono-locus resistance**				**Pyramided resistance**	
	**Pinot Noir**	**BC4 (*Rpv1*)**	**Bianca (*Rpv3-1*)**	**F12P160 (*Rpv12*)**	**Solaris (*Rpv10*)**	**F12P60 (*Rpv3-1; Rpv12*)**	**F12P127 (*Rpv3-1, Rpv3-3; Rpv10*)**
*cis*-piceid	•	•	•	•	•		•
*trans*-piceid	•	•	•	•	•		•
*trans*-resveratrol	•	•	•	•	•		
pallidol			•	•	•		
*trans*-epsilon-viniferin			•		•		
astringin	•			•	•		

The monomer *trans*-resveratrol was identified as significant in mono-locus resistant genotypes and in Pinot Noir comparing inoculated vs. not inoculated samples (Supplementary Table 2); anyhow, in mono-locusresistant genotypes the effect size was higher with a delta d > 1 compared with Pinot Noir. In the mono-locus genotypes the effect size had d > 1 already at 48 hpi with a peak at 96 hpi (Bianca 2.82; F12P160 3.41; Solaris 4.07; BC4 2.02); instead, in Pinot Noir we found an effect size of 1.5 at 96 hpi. As previously reported, *trans*-resveratrol has been identified as a monomer and precursor of active compounds in biotic stress plant defense (Langcake and Pryce, 1977; Jeandet et al., 2002). *tran*s-Piceid, and *cis*-piceid were identified as highly significant both in the mono-locus resistant varieties and in Pinot Noir and in the pyramided genotype F12P127 (Figures 2, 3; Supplementary Figures 1, 2). The effect size values showed an accumulation (d > 1) of these two compounds at 48 hpi and 96 hpi in the resistant genotypes while in Pinot Noir the accumulation has appeared only at 96 hpi (Supplementary Table 3). Astringin was significantly modulated in F12P160 (1.95 at 48 hpi and 1.72 at 96 hpi) and Solaris (1.50 at 48 hpi and 2.85 at 96 hpi) genotypes together with Pinot Noir (1.68 at 96 hpi). The trend of these compounds suggests a role in the response to biotic stress, supported by an early accumulation in the resistant genotypes compared to the susceptible one, but they are probably not directly involved in the defense against the pathogen. We are hypothesizing their modulation confirms that the artificial inoculation of the pathogen was successful. All the identified compounds increased with time after pathogen inoculation and their peak concentration was measured at 48 and 96 hpi (Supplementary Figures 1, 2). The different behavior noticed for the phytoalexins agrees with the reports of Ali et al. (2010, 2012) who found that grapevine-specific phytoalexins can also be produced by the susceptible cultivars upon infection if we consider that at the beginning of the inoculation process the metabolic differences might be acting as the first inducible line of defense. Interesting accumulation was found for pallidol, and *trans*-epsilon-viniferin in all mono-locus genotype, with a significant effect size of these active compounds especially in Solaris at 48 and 96 hpi; these results confirm the importance of dimers biosynthesis and their accumulation in resistance process (Malacarne et al., 2011; Bavaresco et al., 2012; Fröbel et al., 2019).

## Discussion

In time, plants have developed different mechanisms of defense against abiotic and biotic stress. Among these mechanisms, the one between grapevine and *P. viticola* still raises questions concerning the interaction between the pathogen and the metabolism of the plant. It is already known that secondary metabolism has a defensive role against predators, parasites and diseases (Ali et al., 2010), but we shouldn't overlook the role of primary metabolism which, besides controlling the growth, development, and reproduction of plant species, also contributes to the plant defense. It can act as a source of energy, and it can signal molecules to directly or indirectly trigger defense response. This study showed findings of putative biomarkers in primary and secondary metabolism within resistant genotypes, as a defense response to *P. viticola*.

In the present 2-year study, we were able to use four analytical methods to identify and quantify or semi-quantify a large number of metabolites covering the most important compound classes. Among the extensive amount of obtained data, we arbitrarily choose to focus our investigation on the metabolites showing the most significant differences between inoculated vs. not inoculated samples, considering the time points with the criterion described in section Putative Biomarkers of Resistance to *Plasmopara viticola*.

An interesting aspect was observed in the alterations of the metabolism of most of the varieties, but mainly in mono-locus resistant genotypes. Several compounds identified as resistance putative biomarkers had their concentration reduced until 12 h after inoculation, followed by an increase at later time points. A similar reaction to the inoculation with DM was described by Ali et al. (2012) for quercetin-3-O-glucoside, glutamic acid and succinic acid in the resistant genotype Regent (*Rpv3-1*). Although we do not have substantial evidence to explain this behavior, we hypothesize that the pathogen might use these compounds to leak the necessary nutrients from the host cells, right before the activation of the plant defense.

During the infection, the pathogen disturbed the plant metabolism to different degrees. In F12P160 and Solaris, a decrease of the sugars was noticed at 12 h after inoculation, possibly because the pathogen was using them as a source of energy for its proliferation. Although sugars are mainly known in plants as a primary substrate to provide energy during the defense responses, they may also act as signal molecules interacting with the hormonal signaling network to regulate the plant immune system. In their role as plant resistance enhancers, sugars also stimulate the synthesis of flavonoids known as defense-related metabolites (Morkunas and Ratajczak, 2014).

In mono-locus resistant genotypes, the modulation of the metabolites was mainly noticed at 48 hpi and 96 hpi; this clearly indicates that 48 h after inoculation the plant defense mechanisms were active, just like Chitarrini et al. (2017) had noticed in a previous study. Solaris was an exception among the mono-locus resistant genotypes, as it reacted like the pyramided F12P60 genotype, where the modulation of 1-hexanol and benzaldehyde started earlier, between 0 and 12 hpi and reached its peak at 48 or 96 hpi. At the time of the experiments, one *Rpv* resistance gene was described in Solaris (Table 1); the latest report of Possamai et al. (2020) and Vezzulli et al. (2019) reveal the presence of two resistance sources in Solaris (*Rpv3-3* and *Rpv10*), explaining our results and supporting our conclusions. However, additional considerations at the genetic and metabolomics level should be made to fully support that the metabolic changes in Solaris are due to both *Rpv3-3*+*Rpv10*. The earlier activation of the defense response in the pyramided genotypes could be linked to the fact that the pathogen might take around 12 h to germinate and penetrate the leaf, inducing the first metabolic changes due to its colonization (Chitarrini et al., 2017). Another assumption is the presence of two or more resistance sources for *P. viticola* within these genotypes. Besides ensuring a higher degree of resistance and a more stable and durable trait (Merdinoglu et al., 2018; Possamai et al., 2020) it could possibly also trigger a faster reaction against the pathogen.

In plants, lipids are energy storage and signaling compounds. In the defense against environmental factors and pathogens, they function as the structural components of cell membranes, which serve as permeable barriers to the external environment of cells. The accumulation of fatty acids (i.e., stearic acid, erucic acid, palmitic acid, oleic acid+*cis-*vaccenic acids) in plant metabolome after pathogen inoculation indicates their action in the adjustment of membrane fluidity mediated by desaturases and in the intracellular signaling processes (Nishida and Murata, 1996; Laureano et al., 2018) and their profile can be also involved in the protection of photosynthetic machinery in the early stages after the inoculation (Laureano et al., 2018). Thus, due to their role in activating the plant defense response, they are proposed as putative biomarkers. In plants, fatty acids have already been reported as important signaling molecules influencing genes involved in plant-microbe and plant-insect interaction (Savchenko et al., 2010; Walley et al., 2013). In previous experiments we found a decrease in oleic acid+*cis*-vaccenic acid together with other unsaturated fatty acids (16:1, 18:2, and 18:3) at the stage of 24 hpi in *Rpv3* and *Rpv12*-mediated resistance genotypes (Chitarrini et al., 2020). Previous studies report that the deactivation of the desaturase which converts stearic acid to oleic acids leads to an upregulation of salicylic acid (SA)-mediated responses and PR genes, with an inhibition of jasmonic acid (JA)-inducible defenses (Kachroo et al., 2008; Mandal et al., 2012). In our experiment we found an increase of palmitic acid and oleic acid+*cis*-vaccenic acid in F12P60; this situation, which is the opposite of what occurs with the mono-locus genotypes Bianca and F12P160, can be related to a different resistance response of the pyramided genotype.

A large variety of volatile compounds was emitted by the plants after the physiological stress induced by the *P. viticola* (green leaf volatiles, benzenoids, terpenoids, and some unknown compounds). This suggests that the secondary metabolism of the plant was seriously affected to a much higher degree by the pathogen. Green leaf volatiles (GLV) produced by the plant are volatile organic compounds that are released when plants suffer stress at the tissue level. Although the plants release GLVs constantly, they do so to a higher extent under conditions of stress (Hammerbacher and Coutinho, 2019). After pathogen inoculation, we identified two classes of GLVs that were released by plant leaves: alcohols and aldehydes. At physiologically relevant concentrations, a defense role of GLVs is suggested by this study based on their antifungal properties (Fallik et al., 1998). Plants are known to release *trans*-3-hexenal within minutes after they experience pathogen stress, and that such release can last for hours, after which it decreases in concentration as it undergoes enzymatic conversion to 2-hexenal (accumulated in our experiment in Solaris at 48 and 96 hpi) and unsaturated alcohols and esters (Davis et al., 2007). Chitarrini et al. (2017, 2020) had already suggested benzaldehyde as a putative biomarker of resistance, thanks to his role as a promoter of salicylic acid (SA)-mediated defense and its significant accumulation in the plant metabolome at 48 and 96 hpi, with an earlier accumulation in *Rpv12*-mediated resistance compared to the *Rpv3*-mediated one. This confirms our findings, and supports benzaldehyde being a biomarker also in the genotypes Solaris and F12P60, where it was found in significantly increased concentrations. Salicylic acid is the phytohormone precursor of the volatile methyl salicylate found in high concentration in the Bianca resistant genotype; in some plants, it is derived directly from the shikimate pathway in the plastids. Methyl salicylate is known for inducing systemic resistance after the attack of biotrophic organisms, like *P. viticola* (Hammerbacher and Coutinho, 2019).

The resistant grapevine genotypes in our study emitted significantly higher concentrations of terpenoids, both monoterpenes (linalool, neral) and sesquiterpenes (farnesene, (*E*)-nerolidol) than the susceptible genotype Pinot Noir. Hammerbacher and Coutinho (2019) found a positive correlation between an increased plant volatile emission and resistance to *P. viticola*. Algarra Alarcon et al. (2015) found a higher emission of sesquiterpenes and monoterpenes in grapevine genotypes resistant to *P. viticola*. Confirming their role in the fight against the pathogen, the antifungal activity of farnesene, and nerolidol together with ocimene and valencene have been recently tested by Ricciardi et al. (2021) showing a positive effect against the pathogen. In our experiment, farnesene was expressed in high concentrations in three mono-locus resistant genotypes (BC4, Bianca, F12P160) and included in the inclusion criteria for F12P127; linalool was significant only in Bianca genotype and (*E*)-nerolidol and neral were significant in the pyramided genotype F12P60.

The molecules of “unknown4” and “unknown13”, have emerged in the pyramided genotype F12P60. Unfortunately, we do not have enough information about the chemical structure of these compounds; the likelihood of these molecules having a role in plant response to *P. viticola* infection is mentioned in the study by Lazazzara et al. (2018), who described an increase in the abundance of the unknown compounds in resistant genotypes compared to Pinot Noir. Nevertheless, further studies are required to identify the chemical structure and potential roles of these molecules.

Among the flavonoids, epicatechin has been identified in BC4 and, as per the studies of Ali et al. (2012) and Chitarrini et al. (2017); it plays a role in the resistance against pathogens, likely due to its antimicrobial properties.

The stilbenes and stilbenoids identified in mono-locus genotypes and F12P127 are produced through the phenylalanine/polymalonate pathway, and they can have a direct effect on fungal growth and sporulation by slowing down the growth of the pathogen and increasing plant resistance. Fröbel et al. (2019) found a significant induction of phenylalanine ammonium lyase (PAL) and stilbene synthase (STS) genes in *Rpv10* homozygous genotype stating the importance of the quantitative stilbenes produced to stop the pathogen. A recent study by Eisenmann et al. (2019) found that *Rpv3-1*-mediated resistance induces the production of toxic stilbenes and triggers programmed cell death, reducing, but not suppressing, the pathogen growth and development. The accumulation of monomers (*trans*-resveratrol and *cis-* and *trans*-piceid) at the infection site is mainly related to the response to the pathogen inoculation, also found in the susceptible Pinot Noir. Instead, dimers biosynthesis and accumulation, significantly found only in resistant genotypes, can be related to the activity of these compounds against the pathogen (*trans*-epsilon viniferin and pallidol). These dimers have already been identified as markers of resistance representing key defense molecules because they are produced in response to biotic stress (Viret et al., 2018). Moreover, several studies (Del Rio et al., 2004; Atak et al., 2017) found a positive correlation between increased host resistance and an expression of a high content of phenolic compounds; indeed, according to Pezet et al. (2004) our observations demonstrate that stilbenes have significant inhibitory effects on the mobility of *P. viticola* zoospores and on subsequent disease development.

Tables 2, 3 give us a clear identification of the founded markers for each locus.

## Conclusions

This study describes different metabolic responses to the inoculation with *Plasmopara viticola* at various time points post-infection depending on the loci for resistance present in the genotypes.

To our knowledge, this work is the first study to investigate biomarkers present in mono-locus and pyramided-resistant cultivars. We first screen the genotypes with one *Rpv* resistant gene, afterwards we look for genotypes with pyramided resistance to find potential biomarkers associated with different types of resistance to *P. viticola*.

We identified several classes of compounds responsible for the diversification of the resistant cultivars from the susceptible one. We found an interesting modulation on stilbenes and stilbenoids, already known as biomarkers of resistance (dimers active compounds) in the *Vitaceae* and we confirmed the implication of benzaldehyde as a valid biomarker. We found an increase of terpenes emitted by the resistant genotypes confirming their role against the pathogen. Our findings suggest the possibility to test the pathogen inhibition by these VOCs compounds on receiving tissues and the future perspective to use it as a formulation. Interesting accumulations of fatty acids and volatile organic compounds were observed in the pyramided genotype F12P60 which is the variety with the greatest accumulation of potentially active compounds. The high accumulation of the remaining identified metabolites in the resistant genotypes, as compared to the susceptible Pinot Noir, suggests their possible involvement as biomarkers of resistance in a successful defense against *P. viticola*. Further experiments are required to test the putative compounds investigating their effect on infected tissues.

Overall, the results indicate that the way the cultivars responded to pathogen attacks can be linked to genotype and/or to resistant gene differences; however, resistance is not exclusively related to the *Rpv* genes. In our experiment we did not find a strict relation between mono-locus and pyramided response genotypes, even if they have the same *Rpv* genes. We found a higher accumulation of potential resistance biomarkers in Bianca Solaris and F12P60 genotypes. As expected, in the resistance genotypes we identified an Hypersensitive Response (HR) with cell death and necrosis. The pyramided F12P60 genotype that showed interesting metabolites modulation, did not provide any phenotypic evidence of the HR response. Finally, this study provides novel insights into the resistance mechanisms underlying the hybrids-pathogen interaction that could be valuable for the genetic improvement of grapevines.

## Data Availability Statement

Metabolomics raw data are available from MetaboLights (Study Identifier: MTBLS2876, https://www.ebi.ac.uk/metabolights/MTBLS2876)

## Author Contributions

RC, GC, LZ, MS, and UV designed the experiment. MS provided the plant material. RC, GC, and LZ performed the experiment. RC, GC, DŠ, and MR did the extractions and analytical analysis. PF, RC, and GC conducted the data treatment and statistical analysis. RC, GC, and UV prepared the manuscript. UV, GC, MO, and PR supervised the project. All authors discussed the results and implications and commented on the manuscript at all stages.

## Conflict of Interest

The authors declare that the research was conducted in the absence of any commercial or financial relationships that could be construed as a potential conflict of interest.

## Supplementary Material

Supplementary Figure 1 Stilbenes and Stilbenoids meeting the described criteria in mono-locus genotypes; inoculated (Red) and not inoculated (Blue).

Supplementary Figure 2 Stilbenes and Stilbenoids meeting the described criteria in pyramided genotypes; inoculated (Red) and not inoculated (Blue).

Supplementary Table 1 Degree of resistance to *Plasmopara viticola* (OIV-452-leaves) evaluated at 7 days post-inoculation on the first six fully expanded leaves; 1, very low; 3, low; 5, medium; 7, high; 9, very high or total; HR, Hypersensitive Response (necrosis).

Supplementary Table 2 Concentrations of the 22 compounds identified as putative markers of resistance (see section The Effect of Pathogen Inoculation) (VOCs in sheet 1; Lipids in sheet 2 and Polyphenols in sheet 3) and the four stilbenes and stilbenoids involved in the response to the infection (see section Stilbenes and Stilbenoids as Markers) (sheet 3) reported for each genotype and for each year.

Supplementary Table 3 The “d” values of the identified putative biomarkers for the mono-locus and pyramided varieties.
