# Supplementary material for: Mono-Locus and Pyramided Resistant Grapevine Cultivars Reveal Early Putative Biomarkers Upon Artificial Inoculation With Plasmopara viticola
Source: Front Plant Sci. 2021 Jul 1;12:693887. doi: 10.3389/fpls.2021.693887 (PMC8281963; doi:10.3389/fpls.2021.693887)
Supplement: Supplementary Figure 1 — Stilbenes and Stilbenoids meeting the described criteria in mono-locus genotypes; inoculated (Red) and not inoculated (Blue). [file Presentation_1.pptx]

## Slide 1
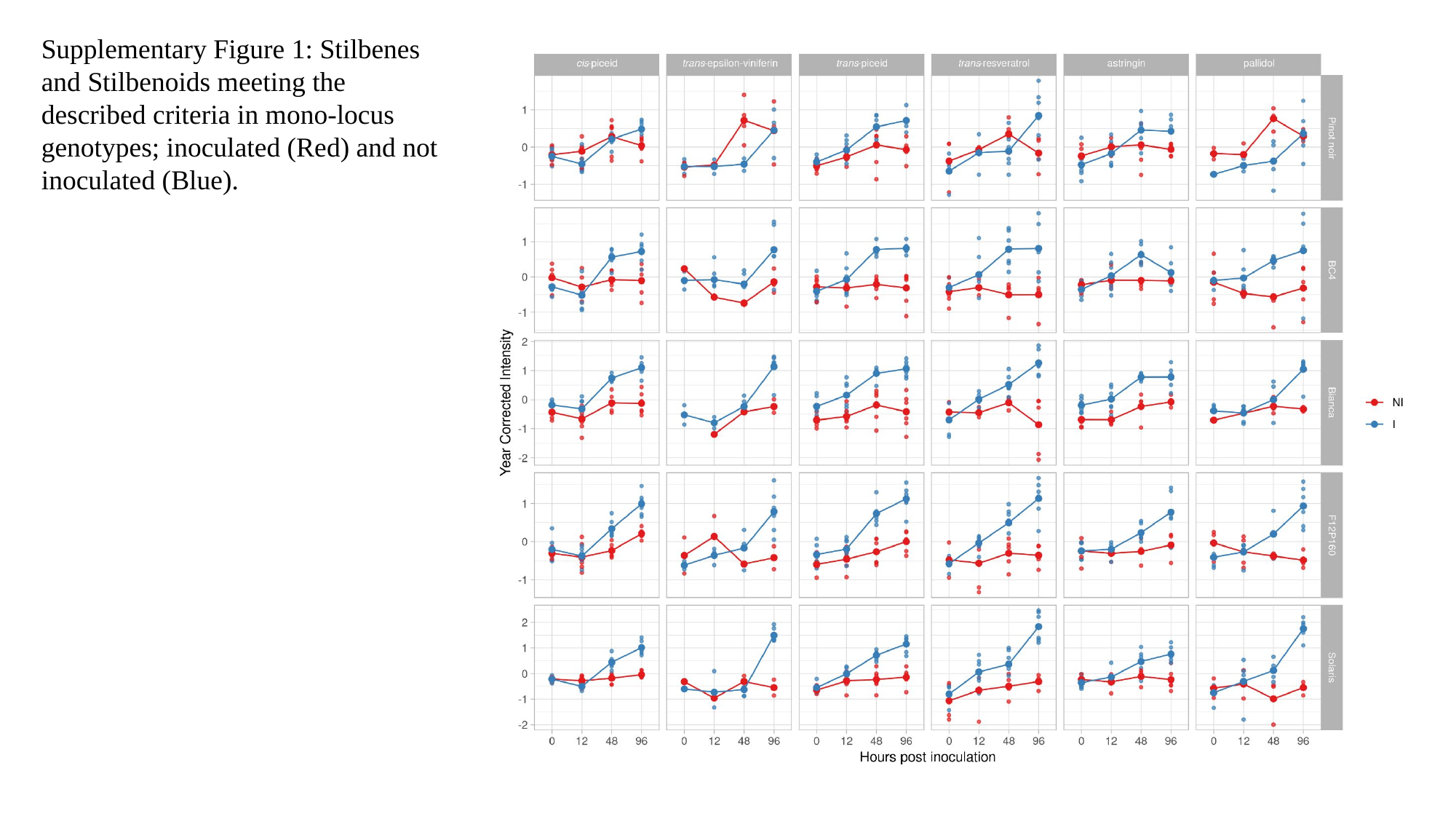

Supplementary Figure 1: Stilbenes and Stilbenoids meeting the described criteria in mono-locus genotypes; inoculated (Red) and not inoculated (Blue).

## Slide 2
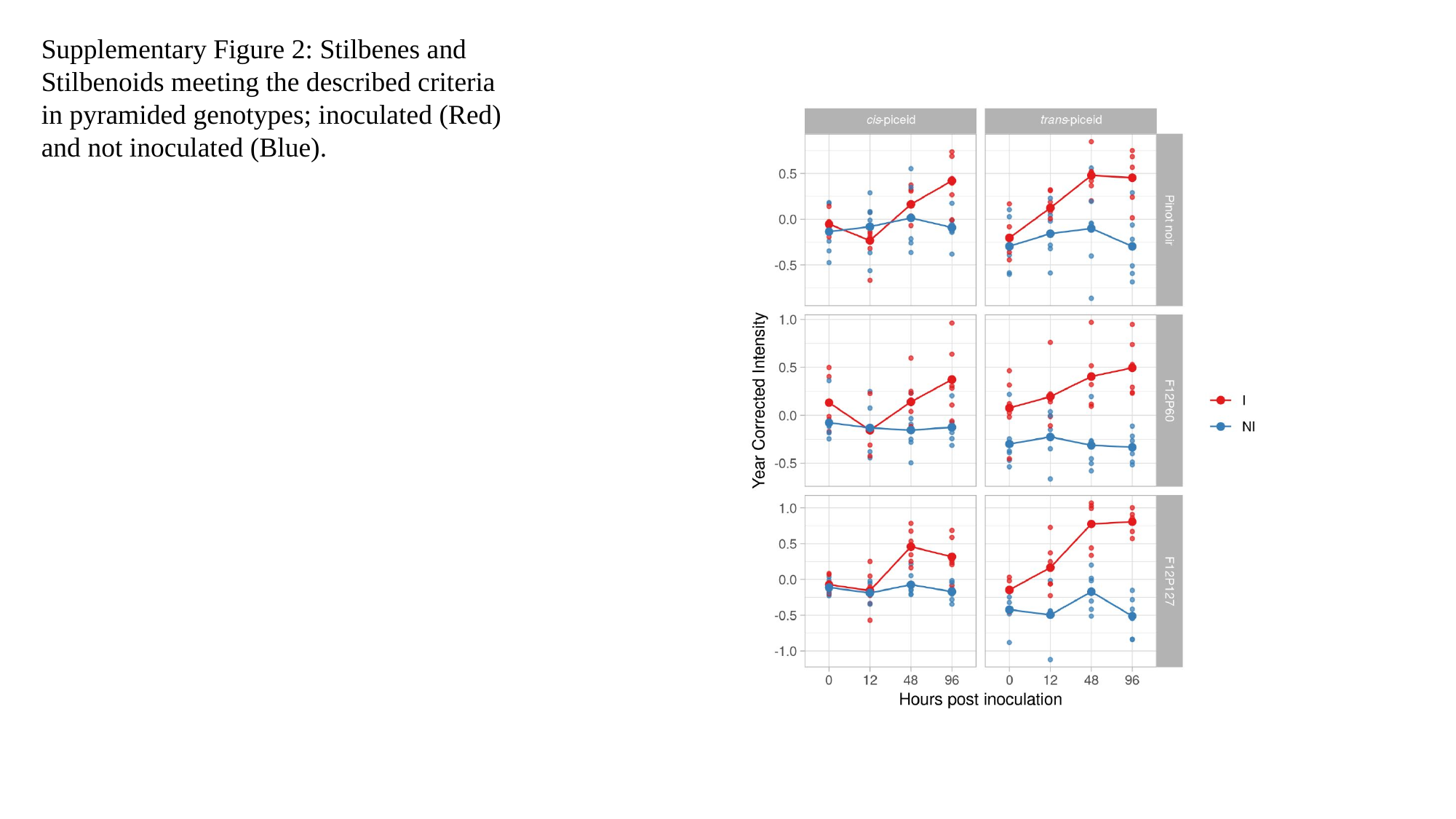

Supplementary Figure 2: Stilbenes and Stilbenoids meeting the described criteria in pyramided genotypes; inoculated (Red) and not inoculated (Blue).
